# Immobilization of Zidovudine Derivatives on the SBA-15 Mesoporous Silica and Evaluation of Their Cytotoxic Activity

**DOI:** 10.1371/journal.pone.0126251

**Published:** 2015-05-05

**Authors:** Dawid Lewandowski, Marta Lewandowska, Piotr Ruszkowski, Anita Pińska, Grzegorz Schroeder

**Affiliations:** 1 Faculty of Chemistry, Adam Mickiewicz University, Poznan, Poland; 2 Faculty of Pharmacy, Poznan University of Medical Sciences, Poznan, Poland; University of Quebect at Trois-Rivieres, CANADA

## Abstract

Novel zidovudine derivatives, able to be covalently conjugated to silica surface, have been obtained and grafted to SBA-15 mesoporous silica. Cytotoxic activity of the hybrid organic-inorganic (zidovudine derivatives-silica) systems against HeLa and KB cell lines has been analyzed. Addition of folic acid had a positive influence on the cytotoxicity. Up to 69% of HeLa and 65% of KB tumor cells growth inhibition has been achieved at low silica concentration used (10 μg/mL).

## Introduction

Mesoporous silicas discovered by Mobil researchers in the early 1990s gave rise to a family of materials that have found numerous applications. From among many morphologically different silicas, the two MCM-41 and SBA-15, have been the most often used. Both represent hexagonal order of mesopores but differ in pore diameters, which for MCM-41 is in the range of 2.0–6.5 nm [1] and for SBA-15–4.0–30.0 nm [2, 3]. MCM-41 is usually used as a carrier for smaller molecules that are packed inside its pores [4, 5], whilst SBA-15 is more suitable for larger ones like proteins [3, 6]. Both can undergo useful surface functionalization, wherein for the SBA-15 a wider variety of molecules is applicable.

Application of mesoporous silicas in the preparation of controlled drug release systems is well known, especially for transportation of anticancer and anti-inflammatory drugs. However, a vast majority of these systems rely on the adsorption properties of anticancer drugs and gate-like structures located on the pore entrances [7] or on surface modifications [8]. Only in very few systems a covalent conjugation of the drug to the silica surface takes place [9].

Physically adsorbed anticancer drugs need to be transported, using mesoporous silica carriers, to the vicinity of target tumor cells and protected from premature release by different stimuli-sensitive moieties. Covalently bound drugs require endocytosis of silica particles by the tumor cells, which already has been reported [10]. The addition of covalently conjugated folic acid enhances particles uptake [10, 11].

Nucleoside analogues, especially 2’,3’-dideoxynucleosides, have found broad application as antiviral [12] and anticancer therapeutics [13–16], designed to mimic natural nucleosides. One of these molecules, which play an important role in anticancer therapeutics is 3’-azido-3’-deoxythymidine (AZT, zidovudine).

AZT has been developed as an antitumor drug, but later it was found to reveal antiretroviral activity and proved to be a proper drug for the treatment of acquired immunodeficiency syndrome (AIDS) [12]. There are also examples of application of AZT as an anticancer agent in therapy of colon cancer, especially in combination with cisplatin, methotrexate and 5-fluorouracil [17, 18].

The mechanism of antiviral or anticancer action of AZT involves its intracellular conversion to its active, 5’-triphosphate form, which is controlled by the cellular enzymes called kinases. The compound 5’-triphosphate is a competitive inhibitor of enzymes involved in the replication process (HIV reverse transcriptase or DNA polymerase) and after incorporation to DNA strand it becomes a chain terminator of the nascent DNA strand due to the lack of 3’-hydroxyl group [19, 20].

In 2001, Sharpless and his co-workers described the concept and criteria for the so-called ‘click’ reactions: versatility, high yield, modularity, readily available starting materials as well as simple reaction conditions. In 2002, Meldal and Sharpless [21, 22] independently, established that the Huisgen 1,3-dipolar cycloaddition reaction between organic azides and terminal alkynes can be efficiently catalyzed by copper(I) ions and can take place at room temperature. As a result, the 1,4-regioisomers of 1,2,3-triazole are formed. ‘Click’ chemistry has been recognized as a valuable synthetic tool in the field of nucleosides and nucleotides since azido and alkyne derivatives are readily accessible [23]. 1,4-Disubstituted 1,2,3-triazole ring is a stable element of the conjugate structure and it is considered as a mimic of *Z*-amide bond. Moreover, it can serve as an additional pharmacophore [24].

To the best of our knowledge, there is no report on zidovudine (AZT) having been successfully grafted onto the silica surface. The aim of this study was to covalently conjugate zidovudine to SBA-15 mesoporous silica surface and analyze the cytotoxic activity of hybrid organic-inorganic systems obtained. All intended aims had been successfully achieved.

## Materials and Methods

### Materials

All reagents and solvents used in the study were purchased from Sigma-Aldrich and used without further purification. SBA-15 mesoporous silica (8–11 nm pore diameter, 600 m^2^*g^-1^ surface area and 1–2 μm particle size) was purchased from ACS Material.

### Synthesis of zidovudine derivatives

**Fig 1:**
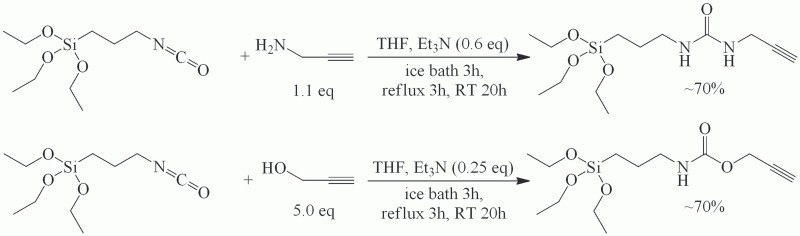
The reaction of synthesis of ‘aza’ and ‘oxy’ intermediates products.

One molar equivalent of (3-isocyanatopropyl)triethoxysilane in THF was placed in an ice bath and an appropriate amount of propargyl compound (1.1 eq. for propargylamine and 5.0 eq. for propargyl alcohol) in the presence of small amounts of triethylamine (0.6 eq. for propargylamine and 0.25 eq. for propargyl alcohol, respectively) was added through a dropping funnel and the mixtures were stirred for 3 h. The amount of propargyl alcohol was much higher due to its weaker nucleophilic properties. While using lower propargyl alcohol:isocyanate ratios, the products of side reactions were detected, especially those of isocyanate self—addition reaction. The next step was to reflux the mixtures for 3 h, allow them to cool to the room temperature and stir overnight. Solvents were evaporated and residues were purified using column chromatography (ethyl acetate:*n*-hexane 3:1) resulting in colorless, viscous liquid and pale yellow solid for ‘oxy’-AZT and ‘aza’-AZT intermediates, respectively (Fig 1). TLC detection was performed using ethyl acetate:*n*-hexane 1:2 as a mobile phase. In both cases the yield was about 70% and the compounds were characterized by electrospray mass spectrometry (Micromass ZQ spectrometer, Waters), melting point measurement (Mel-Temp 1002D), infrared spectroscopy (FT-IR Bruker IFS 66v/S) and ^1^H and ^13^C NMR spectroscopy (VARIAN Mercury 300).

**Fig 2:**
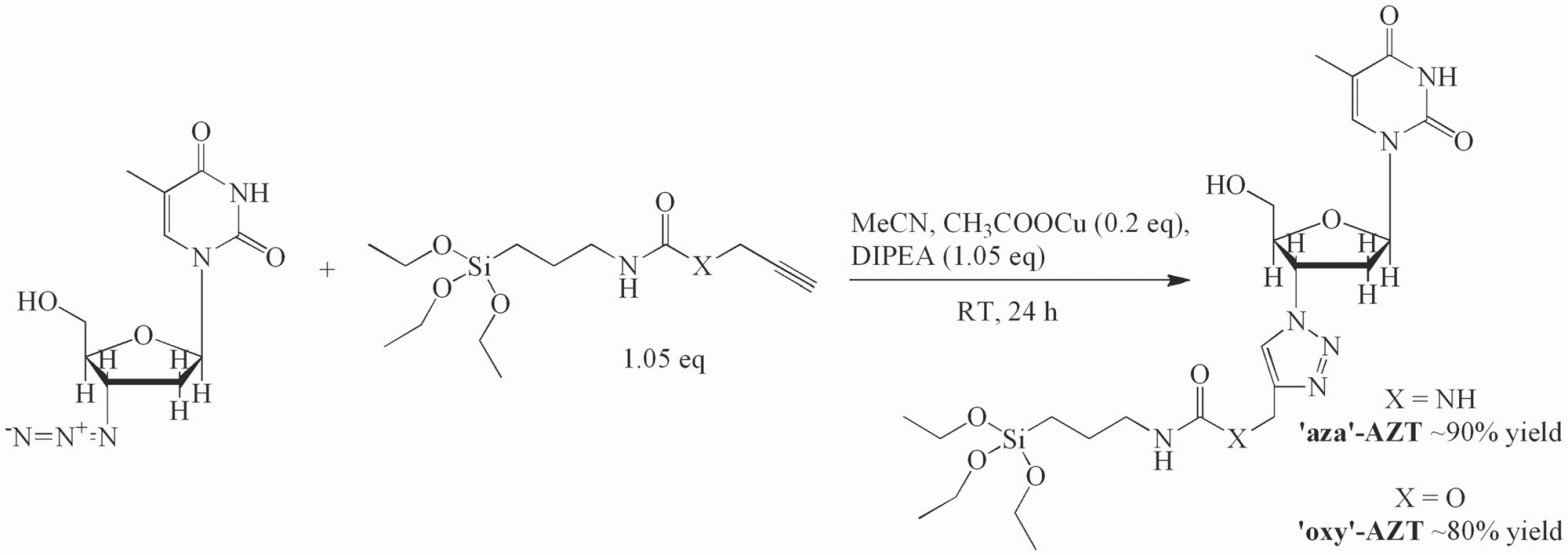
Synthesis reaction of the ‘aza’- and ‘oxy’-AZT derivatives.

In the second step, both intermediates were conjugated with AZT by the1,3-dipolar Huisgen cycloaddition in anhydrous conditions using copper(I) acetate as a catalyst and acetonitrile as a solvent. Exact conditions in both cases were adjusted to achieve the best purity and yield. After the synthesis, both products were purified using column chromatography (methanol:chloroform gradient), which gave pale green (Cu^+^ remnants) and pale orange solids for ‘oxy’-AZT and ‘aza’-AZT (Fig 2). TLC detection was performed using methanol:chloroform 1:10 as a mobile phase. The compounds were characterized by electrospray mass spectrometry (Micromass ZQ spectrometer, Waters), melting point measurement (Mel-Temp 1002D), infrared spectroscopy (FT-IR Bruker IFS 66v/S) and ^1^H and ^13^C NMR spectroscopy (VARIAN Mercury 300).

### Immobilization of zidovudine derivatives on SBA-15 mesoporous silica surface

**Fig 3:**
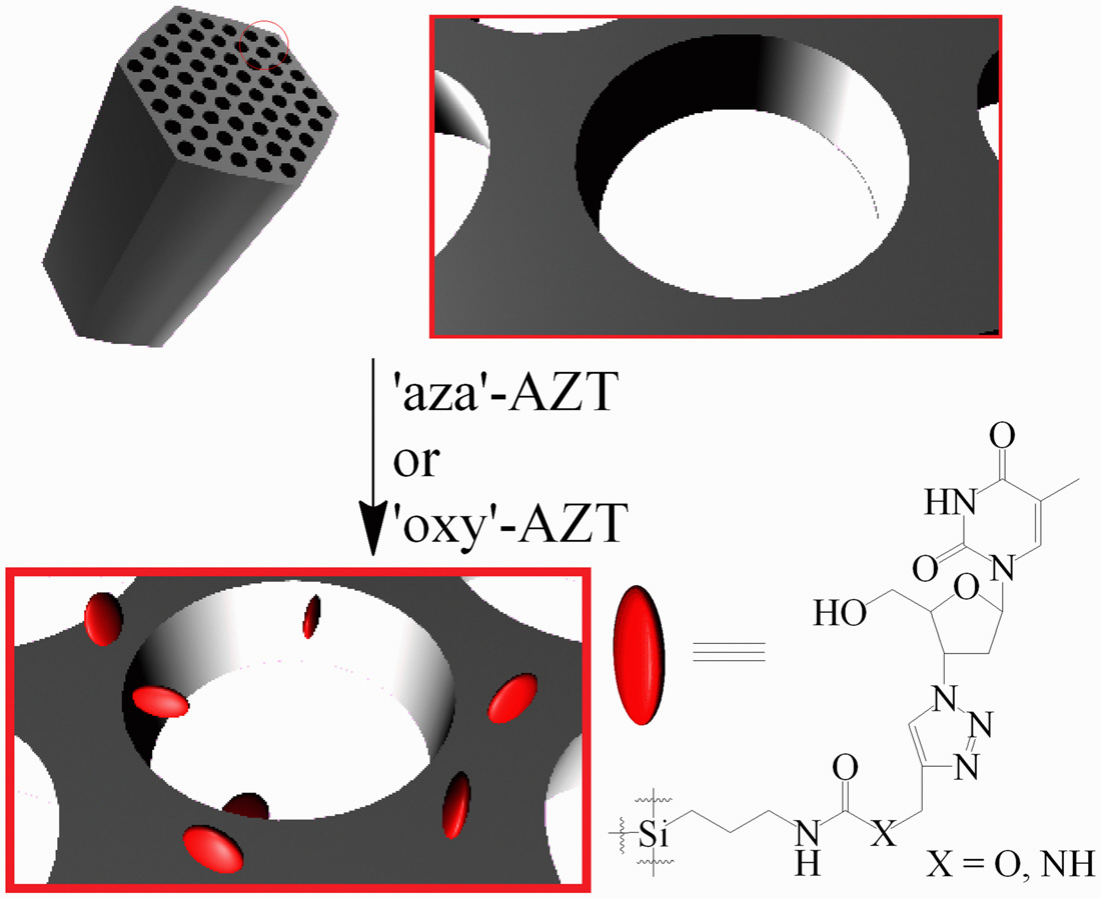
The immobilization of zidovudine derivatives on SBA-15 mesoporous silica surface.

Both derivatives were immobilized on the surface by themselves or together with folic acid. To anchor ‘aza’-AZT and ‘oxy’-AZT on the surface, 70 mg of each of the compounds were dissolved in 10 ml of DMF in separate flasks and 100 mg of SBA-15 silica was added to each flask. Suspensions were stirred at 80°C for 1 h and then allowed to cool to the room temperature and stirred overnight. Modified silica was filtered off, washed thoroughly with DMF and dried. SBA-15+’aza’-AZT and SBA-15+’oxy’-AZT were obtained as white solids (Fig 3). The accurate amounts of both derivatives anchored to the surface were calculated using elemental analysis (elemental analyzer EL III, Vario). The presence of AZT derivatives on the silica surface was additionally confirmed by the IR spectroscopy for 1.0 mg of the silica mixed with 200 mg of KBr and formed into a pellet.

**Fig 4:**
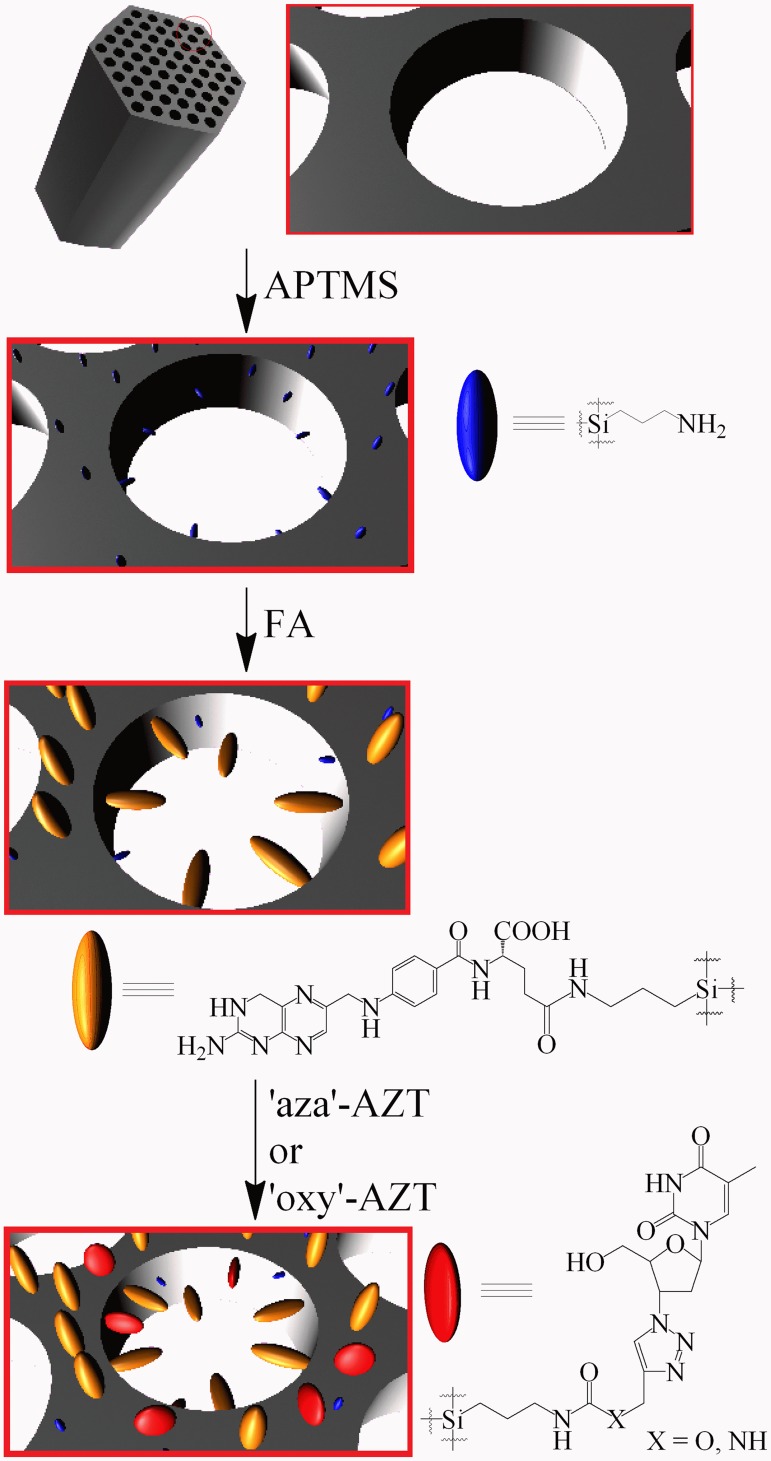
Immobilization of folic acid and both zidovudine derivatives on SBA-15 mesoporous silica surface.

In the second approach 300 mg of SBA-15 silica was suspended in 10 ml of toluene and 30 mg of (3-aminopropyl)trimethoxysilane (APTMS) was added. The mixture was refluxed for 3 h and then allowed to cool to the room temperature and stirred overnight. After that the silica was filtered off, washed with toluene and dried. SBA-15+APTMS as a white solid was obtained. In the next step 50 mg of folic acid (FA) was dissolved in 30 ml of DMF at slightly elevated temperature (40–45°C), an excess of diisopropylcarbodiimide (DIC) (30 mg) was added and, after 15 min, SBA-15+APTMS was suspended. The mixture was stirred for 24 h and then filtered off, washed thoroughly with DMF and dried. SBA-15+APTMS+FA as a pale yellow solid was obtained. In the final step 70 mg of both derivatives, ‘aza’-AZT and ‘oxy’-AZT, were dissolved in 10 ml of chloroform in separate flasks and 150 mg of SBA-15+APTMS+FA was added to each of them. Mixtures were refluxed for 20 min and then allowed to cool and stirred overnight. Both solids were filtered off, washed with chloroform and dried, yielding SBA-15+APTMS+FA+’aza’-AZT and SBA-15+APTMS+FA+’oxy’-AZT (Fig 4). Both products were analyzed using elemental analysis and IR spectroscopy.

### The cytotoxic activity of hybrid materials

Human cancer cells HeLa (cervical cancer cell line) and KB (*carcinoma nasopharynx*) were cultured in RPMI 1640 medium. Each medium was supplemented with 10% fetal bovine serum, 1% L-glutamine and 1% penicillin/streptomycin solution. The cell lines were kept in the incubator at 37°C. The optimal plating density of cell lines was determined to be 5 x 10^4^. All the cell lines were obtained from The European Collection of Cell Cultures (ECACC) supplied by Sigma-Aldrich (catalogue numbers: HeLa cell line-93021013, KB cell line-94050408).

The protein-staining sulforhodamine B (SRB, Sigma-Aldrich) microculture colorimetric assay, developed by the National Cancer Institute (USA) for in vitro antitumor screening was used in this study, to estimate the cell number by providing a sensitive index of total cellular protein content, which is in a linear relationship to the cell density [25]. The monolayer cell culture was trypsinized and the cell count was adjusted to 5 x 10^4^ cells. In each well of the 96 well microtiter plate, 0.1mL of the diluted cell suspension (approximately 10,000 cells) was placed. After 24 hours, when a partial monolayer was formed, the supernatant was washed out and 100 μL of six different silica suspension concentrations were added to the cells in microtiter plates. The tested silicas were suspended in DMSO (20 μM) and the content of DMSO did not exceed 0.1% as this concentration was found to be nontoxic to the cell lines. The cells were exposed to silicas for 72 hours. After that, 25 μL of 50% trichloroacetic acid were added to the wells and the plates were incubated for 1 hour at 4°C. The plates were then washed out with distilled water to remove traces of medium and next dried by air. The air-dried plates were stained with 100 μL SRB and kept for 30 minutes at room temperature. The unbound dye was removed by rapidly washing with 1% acetic acid and then air dried overnight. The optical density was read at 490 nm. All cytotoxicity experiments were performed three times. Cell survival was measured as the percentage absorbance compared to the control (non-treated cells). Zidovudine (Sigma-Aldrich) was used as the internal standard.

## Results and Discussion

### Characterization of synthesized compounds

Both propargyl-containing intermediates were obtained in high purity (full spectra available in the supplementary material):

‘aza’-AZT intermediate—melting point: 35–38°C, ^1^H NMR (300 MHz, CD_3_CN) δ: 0.62–0.67 (2H, m), 1.21–1.25 (9H, t, J = 7.0 Hz), 1.58–1.66 (2H, m), 2.22 (1H, t, J = 2.5 Hz), 3.15–3.21 (2H, dt, J = 6.9 Hz, J = 5.9 Hz), 3.80–3.85 (6H, q, J = 7.0 Hz), 3.97–4.00 (2H, dd, J = 2.5 Hz, J = 5.5 Hz), 5.08–5.15 (2H, m), ^13^C NMR (75 MHz, CDCl_3_) δ: 7.5, 18.2, 23.5, 30.0, 42.8, 58.4, 70.9, 80.8, 157.9, FT-IR: 3330 cm^-1^ (N-H ν_st_), 3250 cm^-1^ (≡C-H ν_st_), 2975, 2925 and 2885 cm^-1^ (aliphatic C-H ν_st_), 2115 cm^-1^ (C≡C ν_w_), 1625 cm^-1^ (urea C = O ν_st_), 1570 cm^-1^ (N-H δ_st_), 1080 cm^-1^ (Si-O ν_vs_), ESI MS *m/z* [M-C_2_H_5_O]^+^ 257.3, [M+Na]^+^ 325.3, [M+K]^+^ 341.3.

‘oxy’-AZT intermediate—melting point: below -25°C, ^1^H NMR and ^13^C NMR data can be found in literature [26], FT-IR: 3340 cm^-1^ (N-H ν_st_), 3315 cm^-1^ (≡C-H ν_st_), 2975, 2930 and 2890 cm^-1^ (aliphatic C-H ν_st_), 2130 cm^-1^ (C≡C ν_w_), 1715 cm^-1^ (carbamate C = O ν_st_), 1535 cm^-1^ (N-H δ_st_), 1235 cm^-1^ (C-O ν_st_), 1080 cm^-1^ (Si-O ν_vs_), ESI MS *m/z* [M-C_2_H_5_O]^+^258.2, [M+Na]^+^ 326.3, [M+K]^+^ 342.3.

‘aza’-AZT final product—melting point: 85–88°C, ^1^H NMR (300 MHz, CD_3_CN) δ: 0.53–0.58 (2H, m), 1.14–1.19 (9H, t, J = 7.0 Hz), 1.44–1.53 (2H, m), 1.85–1.86 (3H, d, J = 1.2 Hz), 2.60–2.68 (1H, m), 2.76–2,83 (1H, m), 3.02–3.08 (2H, dt, J = 6.9 Hz, J = 6.0 Hz), 3.47–3.51 (1H, t), 3.66–3.72 (1H, m), 3.74–3.84 (7H, m), 4.27–4.31 (1H, dt, J = 3.1 Hz, J = 5.4 Hz), 4.31–4.33 (2H, d, J = 5.8 Hz), 5.11–5.15 (1H, t, J = 5.7 Hz), 5.26–5.31 (1H, dt, J = 5.4 Hz, J = 8.5 Hz), 5.36–5.41 (1H, t, J = 5.7 Hz), 6.38–6.42 (1H, t, J = 6.6 Hz), 7.65–7.66 (1H, q, J = 1.2 Hz), 7.75 (1H, s), 9.10 (1H, s), ^13^C NMR (75 MHz, CDCl_3_) δ: 7.6, 12.4, 18.2, 23.6, 35.5, 37.9, 43.0, 58.4, 59.4, 61.3, 85.2, 86.4, 110.9, 122.7, 137.1, 146.5, 150.8, 158.9, 164.6, FT-IR: 3320 cm^-1^ (N-H ν_m_), 3065 cm^-1^ (= C-H ν_m_), 2975, 2925 and 2890 cm^-1^ (aliphatic C-H ν_st_), 1700 cm^-1^ (pyrimidine C = O ν_st_ and urea C = O ν_st_ as a distortion of this signal), 1560 cm^-1^ (N-H δ_st_), 1275 cm^-1^ (sugar C-O ν_m_), 1080 cm^-1^ (Si-O ν_vs_), ESI MS *m/z* [M+Na]^+^ 592.4, [2M+Na]^+^1161.7.

‘oxy’-AZT final product—melting point: 56–59°C, ^1^H NMR (300 MHz, CD_3_CN) δ: 0.53–0.59 (2H, m), 1.14–1.19 (9H, t, J = 7.0 Hz), 1.47–1.56 (2H, m), 1.85–1.86 (3H, d, J = 1.2 Hz), 2.61–2.69 (1H, m), 2.77–2.84 (1H, m), 3.03–3.09 (2H, q, J = 6.8 Hz), 3.31 (1H, s), 3.68–3.73 (1H, dd, J = 3.1 Hz, J = 12.3 Hz), 3.74–3.84 (7H, m), 4.29–4.33 (1H, dt, J = 3.1 Hz, J = 5.4 Hz), 5.10 (2H, s), 5.29–5.34 (1H, dt, J = 5.3, J = 8.5 Hz), 5.61–5.67 (1H, t, broad signal), 6.38–6.43 (1H, t, J = 6.6 Hz), 7.64–7.65 (1H, q, J = 1.2 Hz), 7.88 (1H, s), 9.01 (1H, s), ^13^C NMR (75 MHz, CDCl_3_) δ: 7.5, 12.4, 18.2, 23.1, 37.6, 43.4, 57.6, 58.4, 59.1, 61.2, 85.2, 87.6, 111.0, 124.2, 137.5, 143.8, 150.5, 156.2, 164.2, FT-IR: 3330 cm^-1^ (N-H ν_m_), 3065 cm^-1^ (= C-H ν_m_), 2975, 2930 and 2890 cm^-1^ (aliphatic C-H ν_st_), 2105 cm^-1^ (very weak—probably unreacted AZT), 1700 cm^-1^ (pyrimidine C = O ν_st_ and carbamate C = O ν_st_), 1535 cm^-1^ (N-H δ_st_), 1275 cm^-1^ (sugar C-O ν_m_), 1080 cm^-1^ (Si-O ν_vs_), ESI MS *m/z* [M+Na]^+^ 593.3, [2M+Na]^+^ 1163.5.

### Characterization of modified silica obtained

**Fig 5:**
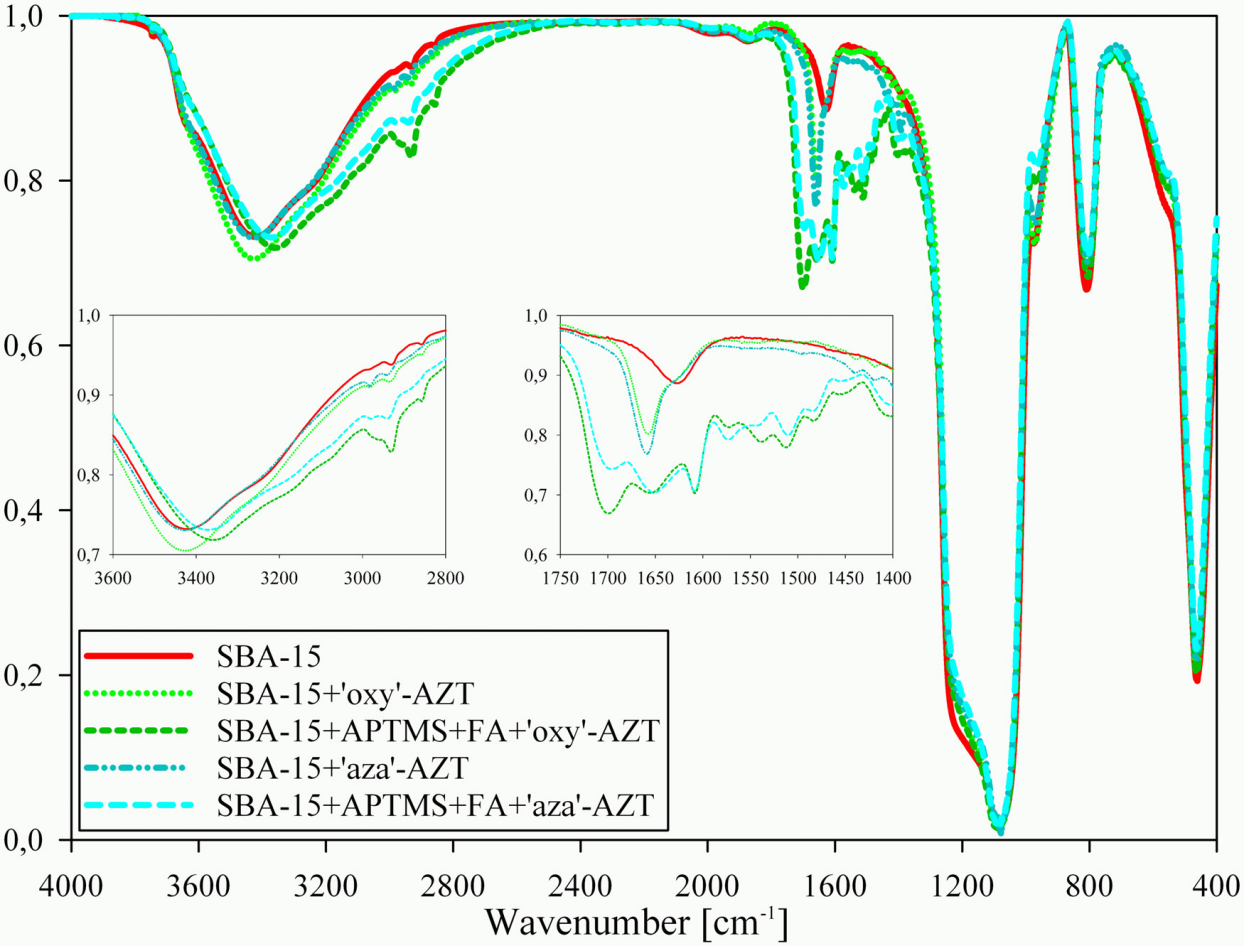
IR spectra of all hybrid systems obtained and pure SBA-15 mesoporous silica for comparison.

Mean amounts of nitrogen and carbon in SBA-15+’aza’-AZT equal to, respectively, 0.471% and 1.379% permitted calculation of the total amount of ‘aza’-AZT on the silica surface. As the content of carbon is slightly dependent on the number of Si-O-Si links between ‘aza’-AZT and silica surface, the nitrogen percentage is more reliable and gives 2.735±0.185% mass percentage of ‘aza’-AZT, that is 48.1±3.3 μmol of ‘aza’-AZT per 1g of modified silica. Similar calculations for SBA-15+‘oxy’-AZT, with 0.374% of nitrogen and 1.247% of carbon content, lead to 2.538±0.058% mass percentage or 44.5±1.0 μmol of ‘oxy’-AZT per 1 g of modified silica. The IR spectra (Fig 5) indicate the presence of both zidovudine derivatives, which can be proved by the presence of C = O amide stretching signal at 1659 cm^-1^, other signals are invisible, because of the low derivative content. The shift of the signal assigned to the C = O bond can be connected with the immobilisation process. Moreover, this signal in the spectra of zidovudine, according to the literature [27–31], can be found between 1700 and 1650 cm^-1^.

IR spectra of the systems containing folic acid are more complicated. However, the main signals can still be assigned, the signal at1654 cm^-1^ is assigned to the stretching of C = O amide from zidovudine, the signals at 1698 and 1609 cm^-1^ come from C = O carboxyl and C = O amide groups of folic acid respectively. The shift of the peak assigned to the stretching of O-H from 3425–3420 cm^-1^ for the silicas without folic acid to 3370–3360 cm^-1^ for both systems with folic acid is thus also related to the presence of folic acid. Elemental analysis results for both modified silicas are 3.428% of nitrogen and 9.019% of carbon for SBA-15+APTMS+FA+’oxy’-AZT and 3.703% of nitrogen and 9.237% of carbon for SBA-15+APTMS+FA+’aza’-AZT. They are difficult to interpret because of the number of steps in the synthesis. Nevertheless, the mass increase correlates well with the elemental analysis results for simple SBA-15+‘aza’-AZT and SBA-15+’oxy’-AZT systems and following this line of reasoning it can be calculated that in SBA-15+APTMS+FA+’aza’-AZT and SBA-15+APTMS+FA+’oxy’-AZT systems the amounts of zidovudine derivatives are slightly above 100 μmol per 1 g of modified silica, which is about twice as much as for simple SBA-15+’aza’-AZT and SBA-15+’oxy’-AZT systems. Small amounts of APTMS used in the first step of the synthesis left enough free space on the surface to anchor both derivatives, moreover the use of another solvent (of a lower dielectric constant to facilitate its penetration into the silica [32]) brought a positive effect on their total amounts.

### The cytotoxic activity

**Fig 6:**
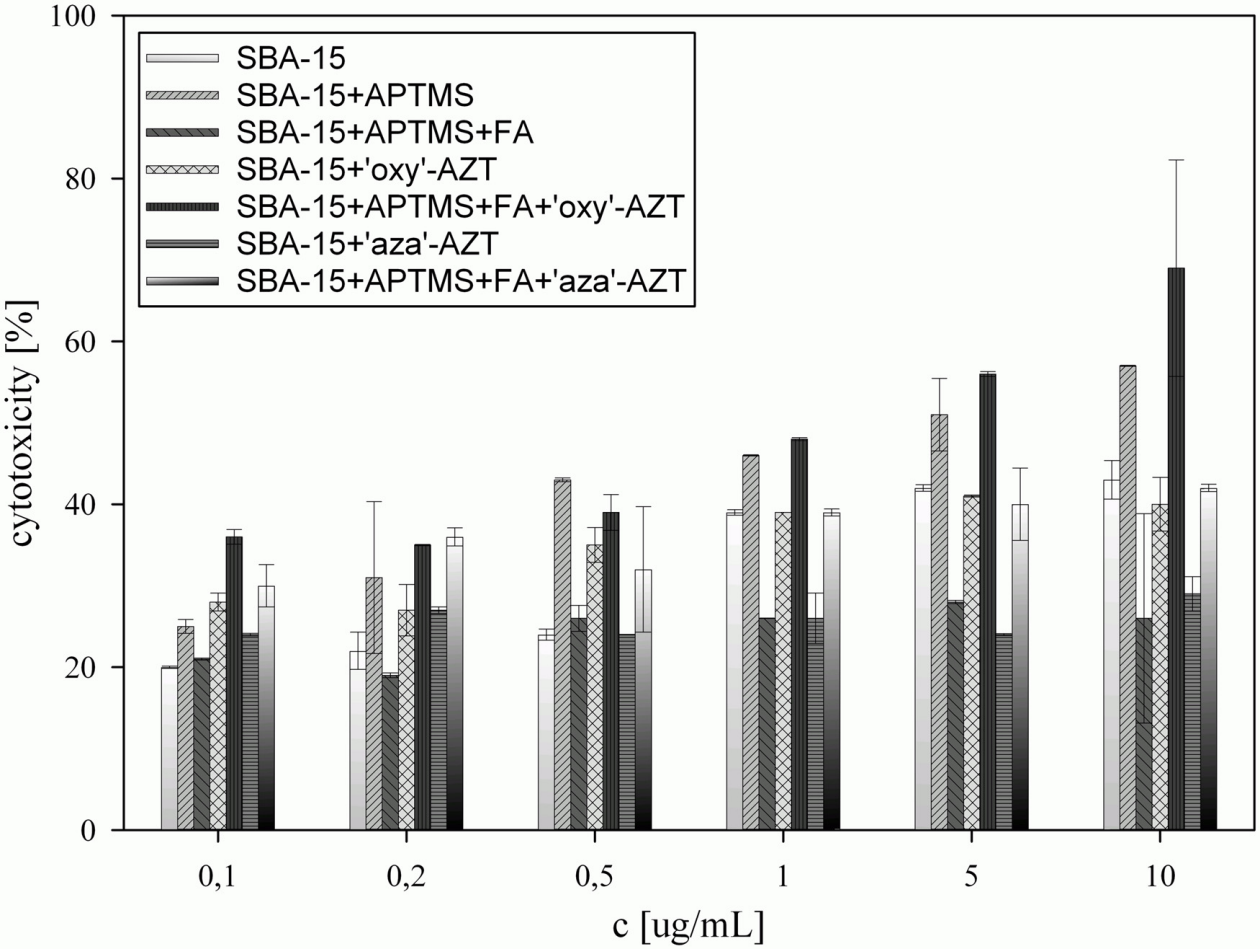
Cytotoxic activity against HeLa cell line, calculated for all samples tested.

**Fig 7:**
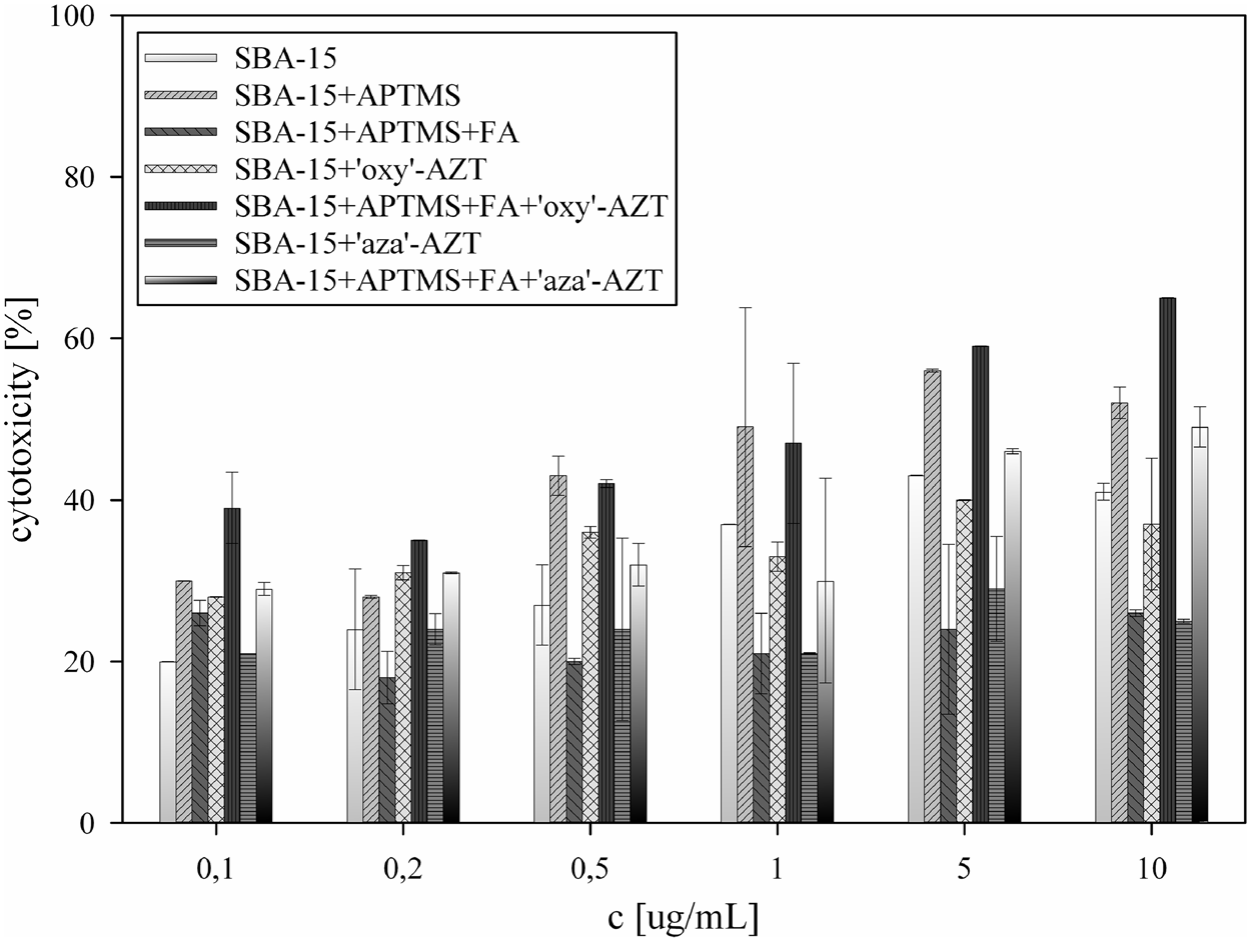
Cytotoxic activity against KB cell line, calculated for all samples tested.

All analyzed solids showed concentration-dependent cytotoxic activity against HeLa and KB cell lines (Fig 6 and Fig 7). Introduction of amino groups on the silica surface greatly increased its activity, which is in agreement with the results observed for pure, organic compounds [33–35].

Covering the surface with folic acid had ambivalent influence. On the one hand, modification of the amino groups with folic acid causes a decrease in the activity of SBA-15+APTMS+FA, because the access to primary amino groups is blocked with folic acid molecules. On the other hand, the addition of folic acid molecules to both systems containing zidovudine derivatives, significantly increases their activity, promoting the interactions between silica particles and cell membrane, as KB and HeLa cell lines are known to be FR-positive [36]. Higher cell growth inhibition observed for SBA-15+APTMS+FA+’oxy’-AZT in comparison with the result for SBA-15+APTMS+FA+’aza’-AZT may be due to the fact that carbamate group in the ‘oxy’ derivative is more susceptible to hydrolysis than the urea-like one in the ‘aza’ derivative, that means it can release more zidovudine molecules from the surface. Cytotoxic activities obtained, especially that of SBA-15+APTMS+FA+’oxy’-AZT, are comparable to those of pure zidovudine(IC_50_ = 3.12 μg/mL for KB and 2.28 μg/mL for HeLa cell line), but at much lower zidovudine concentration. High deviations of the results obtained for some of the samples are a result of sedimentation in silica suspensions.

## Conclusions

Two novel zidovudine derivatives have been obtained and characterized. Both of them have been easily introduced onto the SBA-15 mesoporous silica surface using the grafting technique. Cytotoxic activity against HeLa and KB cell lines of all solids obtained has been evaluated. The addition of folic acid on the silica surface enhances the activity of the derivatives studied due to the interaction with folate receptors. The values of cell growth inhibition obtained for SBA-15+APTMS+FA+’oxy’-AZT are relatively high and equal to 69% and 65% for HeLa and KB tumor cells. The results of this study can be a basis for further attempts at covalent conjugation of cytotoxic compounds to the silica surface.

## Supporting Information

S1 Spectrum Full ESI MS spectra of ‘aza’- and ‘oxy’-AZT intermediates. (TIF)

S2 Spectrum Full FT-IR spectra of ‘aza’- and ‘oxy’-AZT intermediates. (TIF)

S3 Spectrum ^1^H NMR spectrum of ‘aza’-AZT intermediate. (TIF)

S4 Spectrum ^13^C NMR spectrum of ‘aza’-AZT intermediate. (TIF)

S5 Spectrum Full ESI MS spectra of ‘aza’- and ‘oxy’-AZT final products. (TIF)

S6 Spectrum Full FT-IR spectra of ‘aza’- and ‘oxy’-AZT final products. (TIF)

S7 Spectrum ^1^H NMR spectrum of ‘aza’-AZT final product. (TIF)

S8 Spectrum ^13^C NMR spectrum of ‘aza’-AZT final product. (TIF)

S9 Spectrum ^1^H NMR spectrum of ‘oxy’-AZT final product. (TIF)

S10 Spectrum ^13^C NMR spectrum of ‘oxy’-AZT final product. (TIF)
