# Supplementary figures and images for: Immobilization of Zidovudine Derivatives on the SBA-15 Mesoporous Silica and Evaluation of Their Cytotoxic Activity
Source: PLoS One. 2015 May 5;10(5):e0126251. doi: 10.1371/journal.pone.0126251 (PMC4420263; doi:10.1371/journal.pone.0126251)

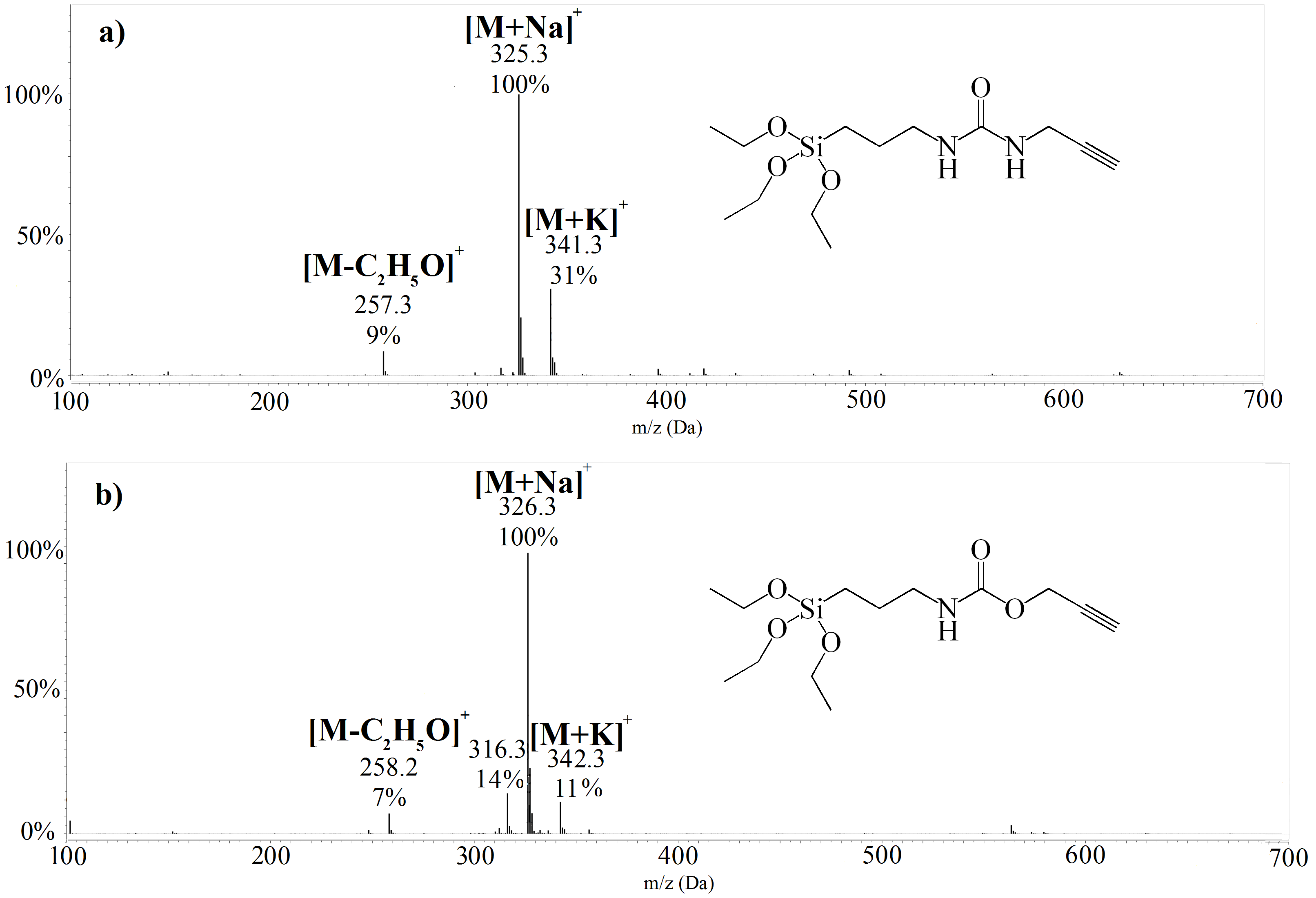

Supplement: S1 Spectrum — (TIF) [file pone.0126251.s001.tif]

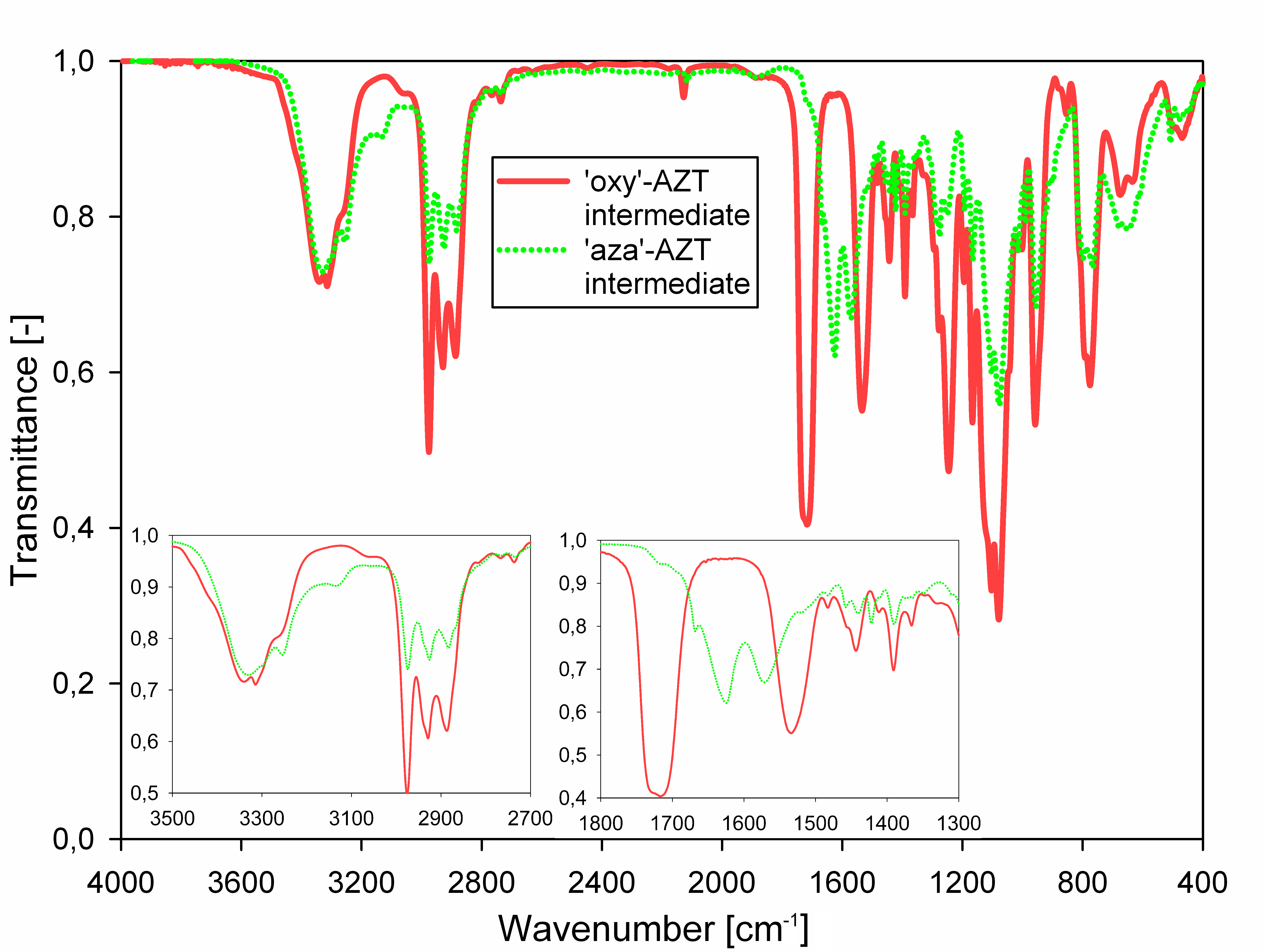

Supplement: S2 Spectrum — (TIF) [file pone.0126251.s002.tif]

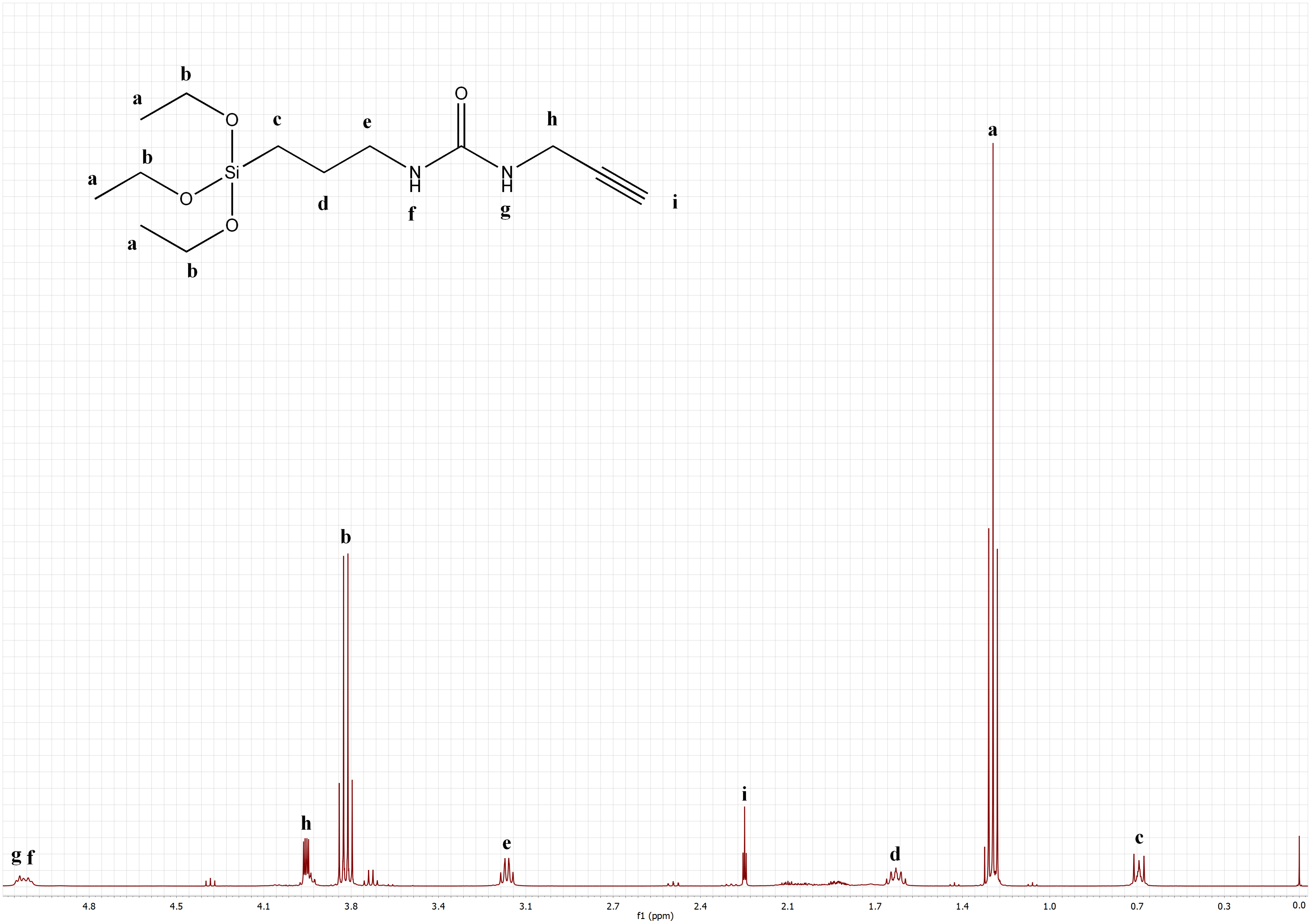

Supplement: S3 Spectrum — (TIF) [file pone.0126251.s003.tif]

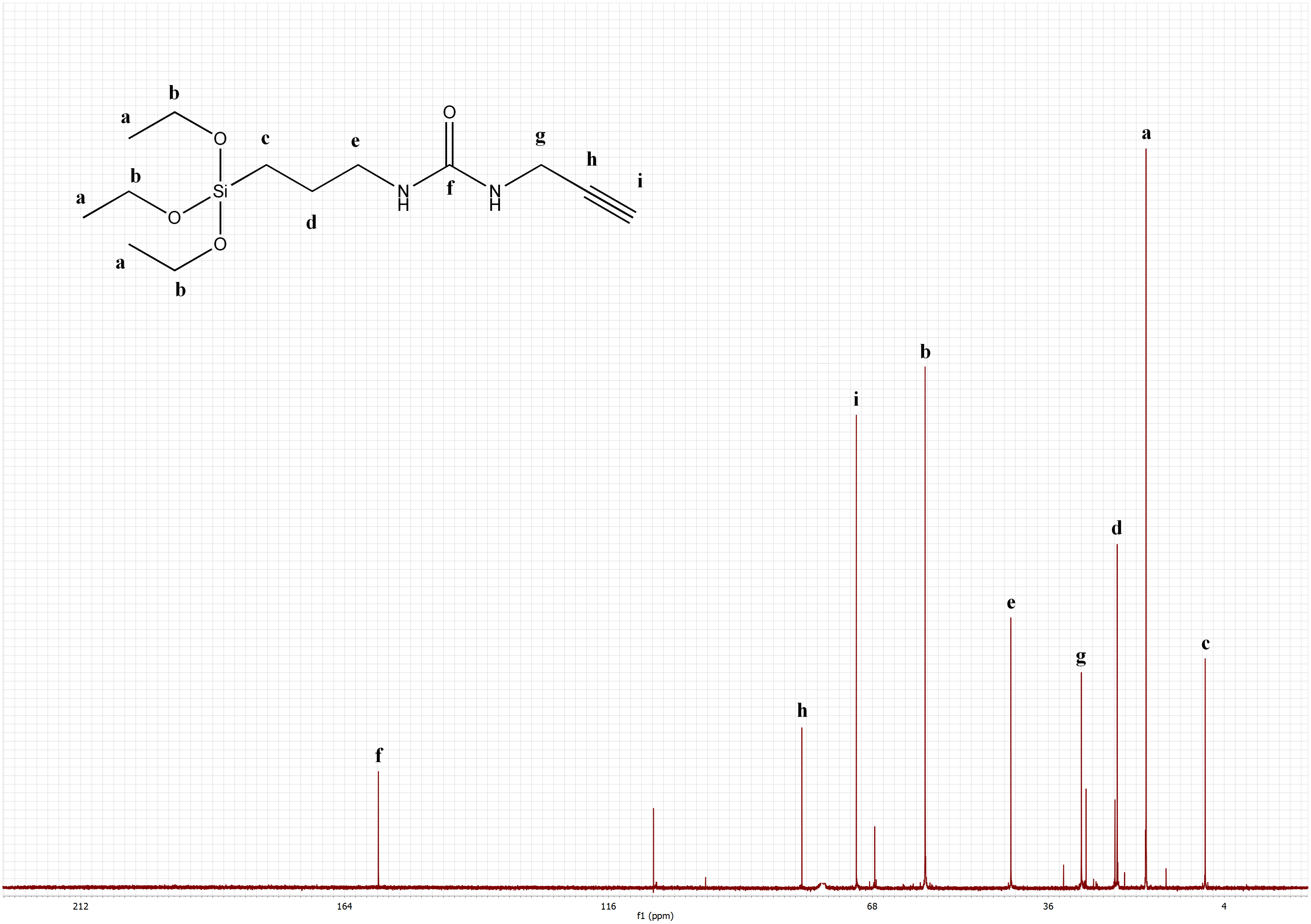

Supplement: S4 Spectrum — (TIF) [file pone.0126251.s004.tif]

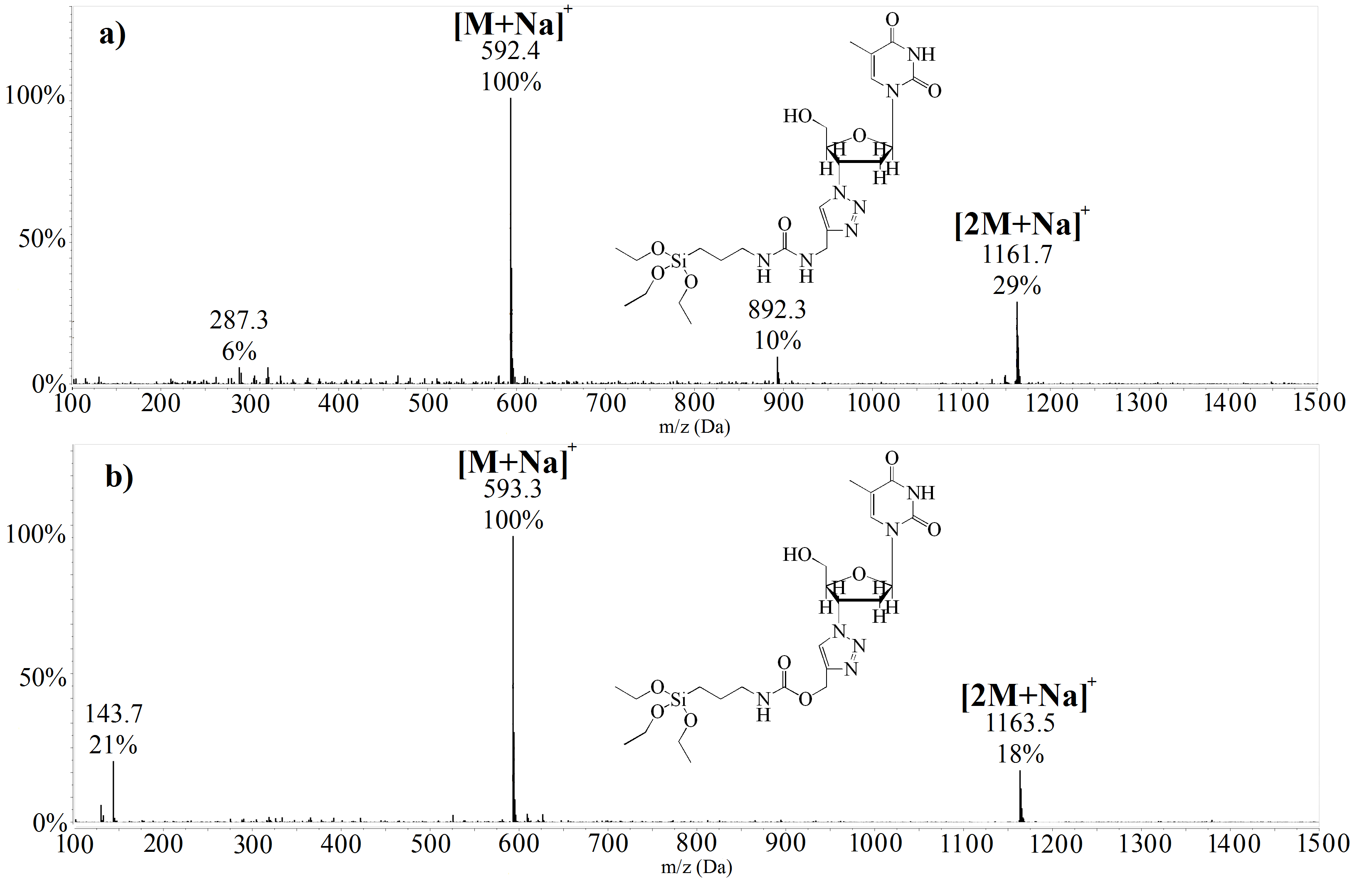

Supplement: S5 Spectrum — (TIF) [file pone.0126251.s005.tif]

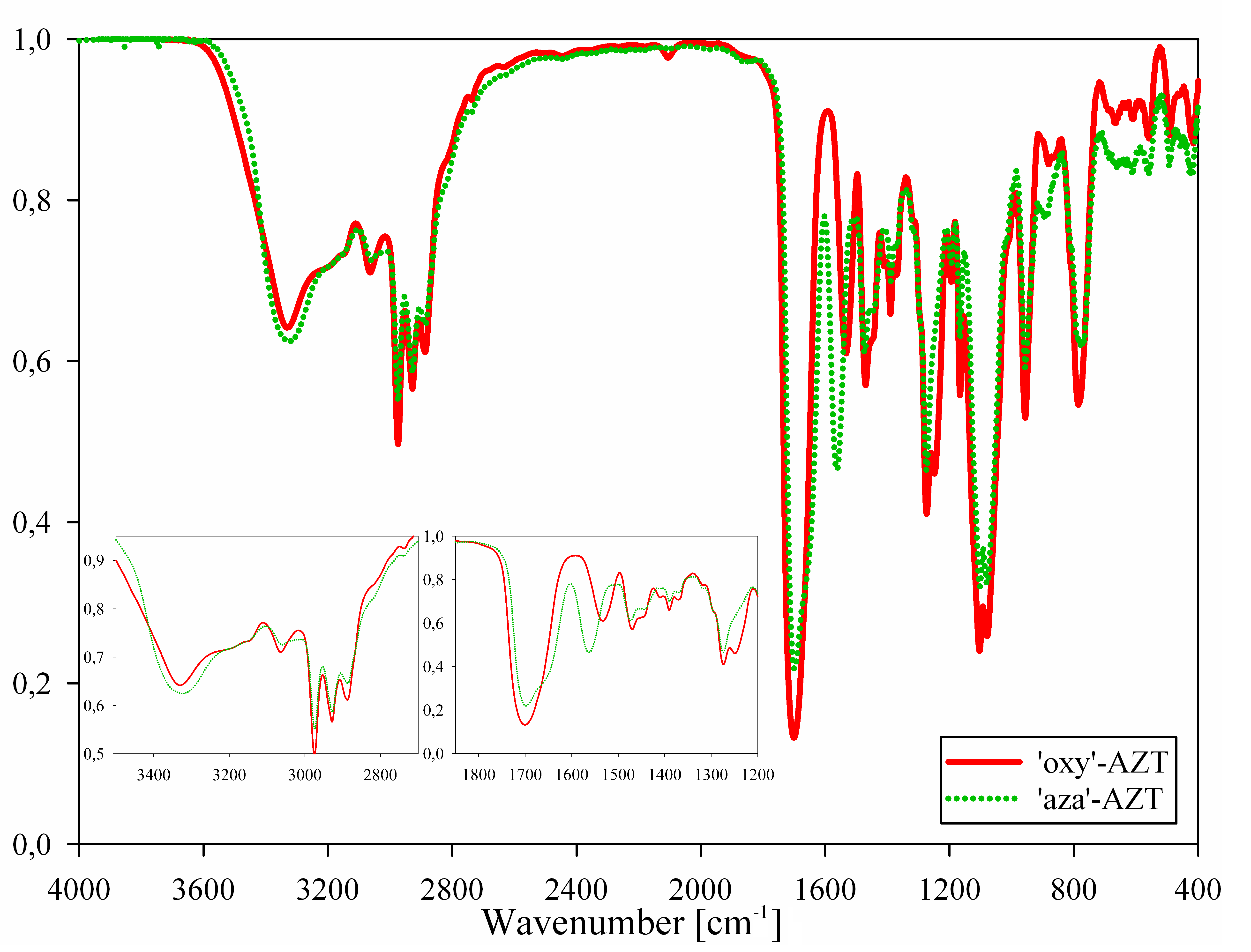

Supplement: S6 Spectrum — (TIF) [file pone.0126251.s006.tif]

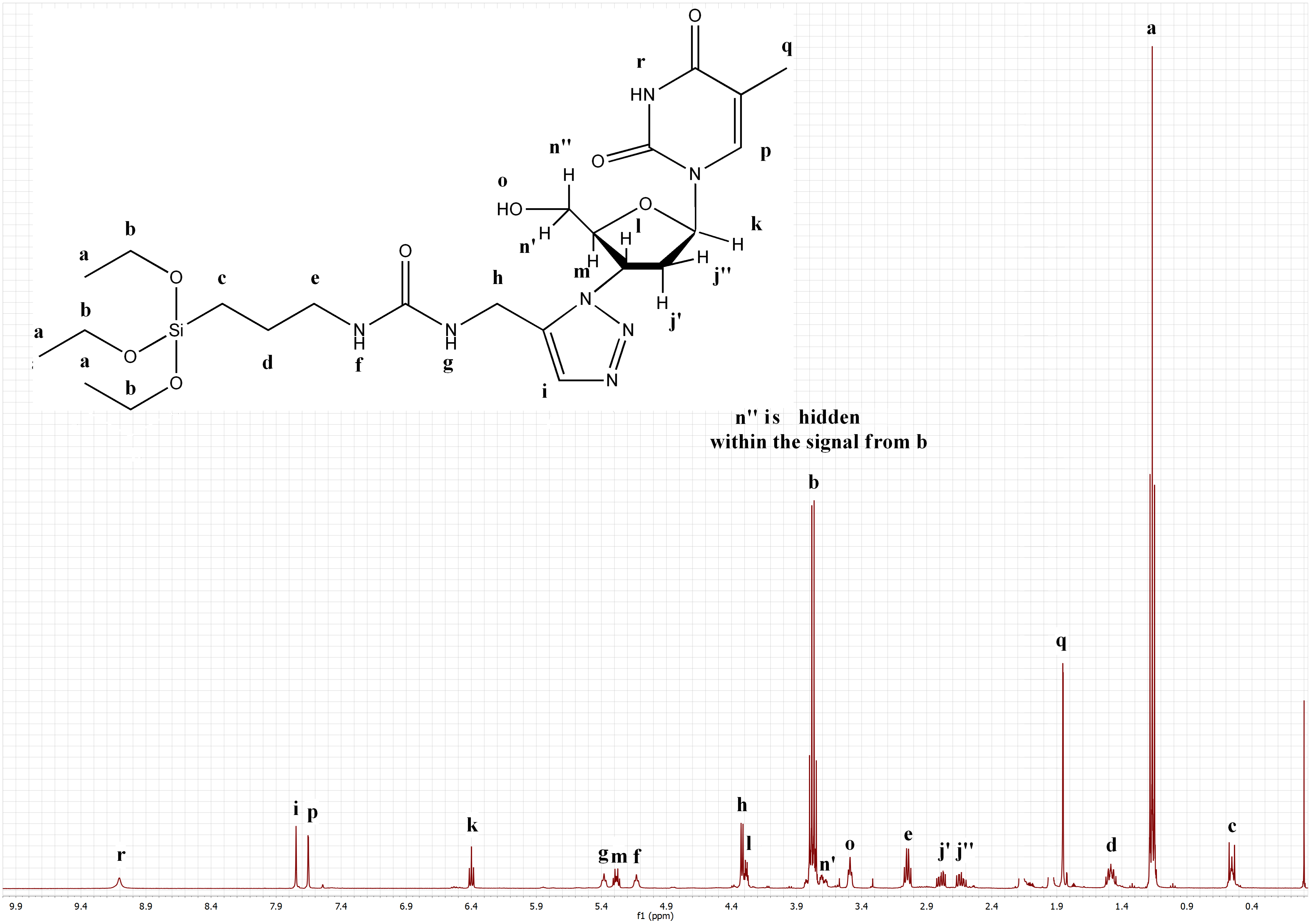

Supplement: S7 Spectrum — (TIF) [file pone.0126251.s007.tif]

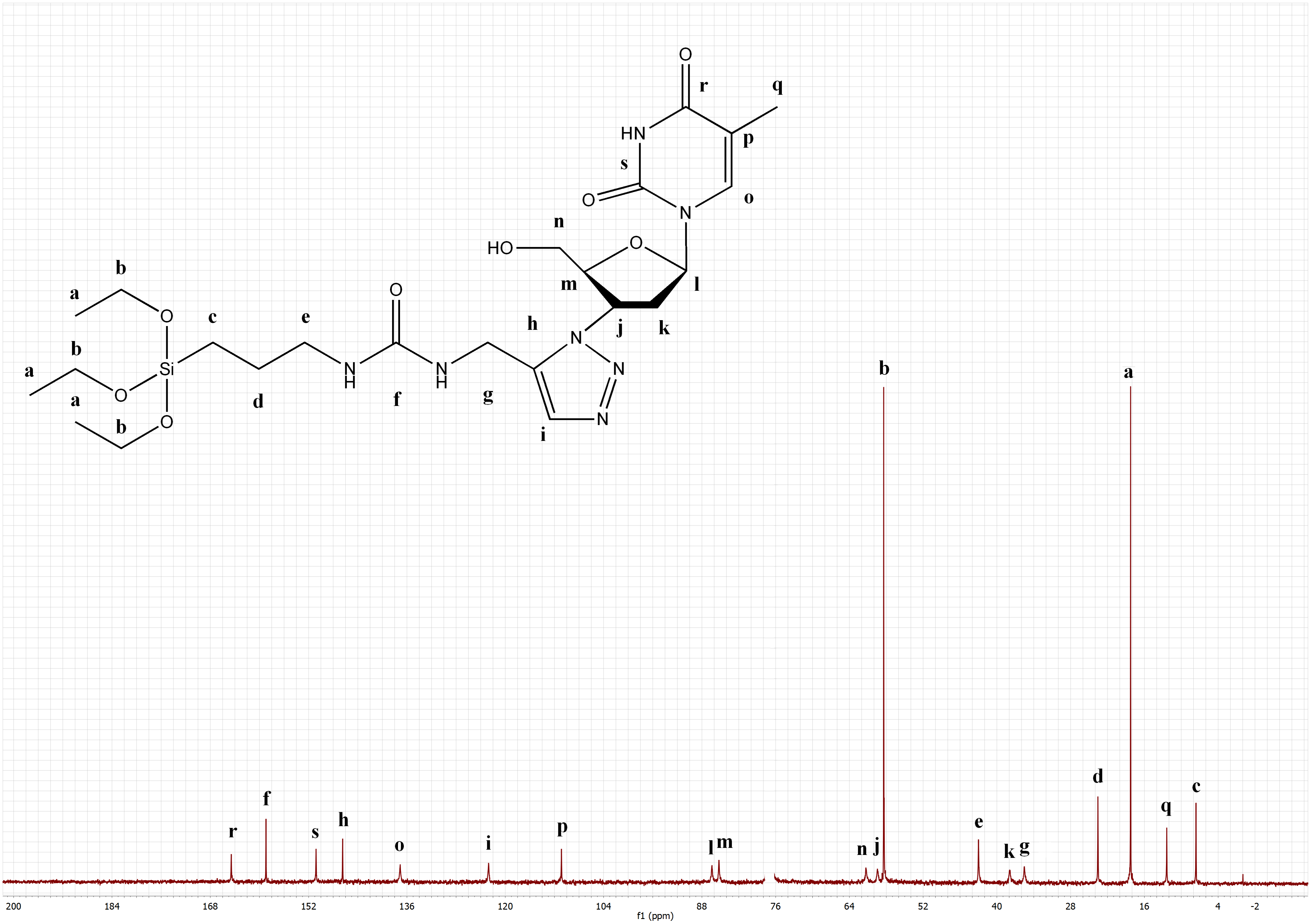

Supplement: S8 Spectrum — (TIF) [file pone.0126251.s008.tif]

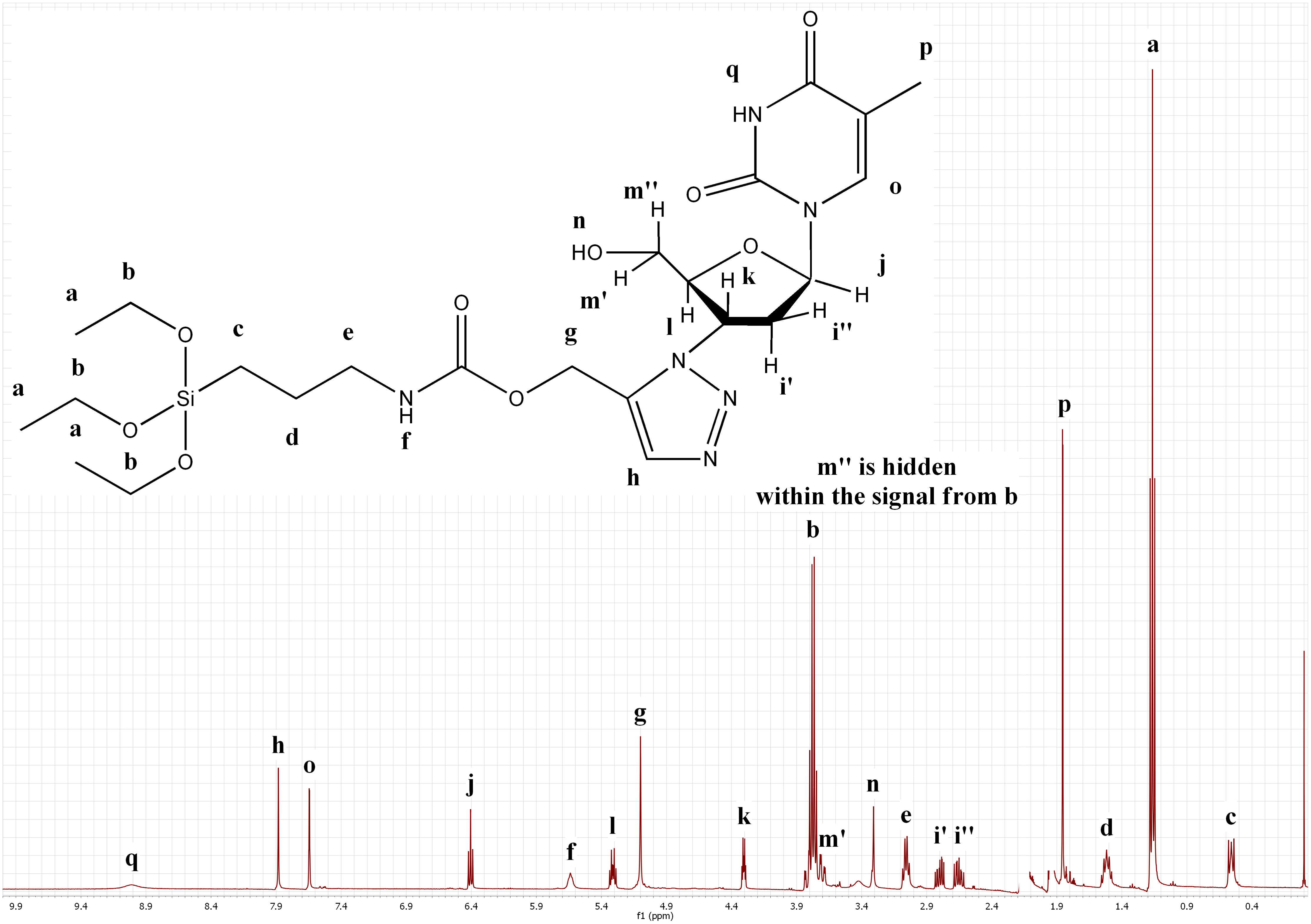

Supplement: S9 Spectrum — (TIF) [file pone.0126251.s009.tif]

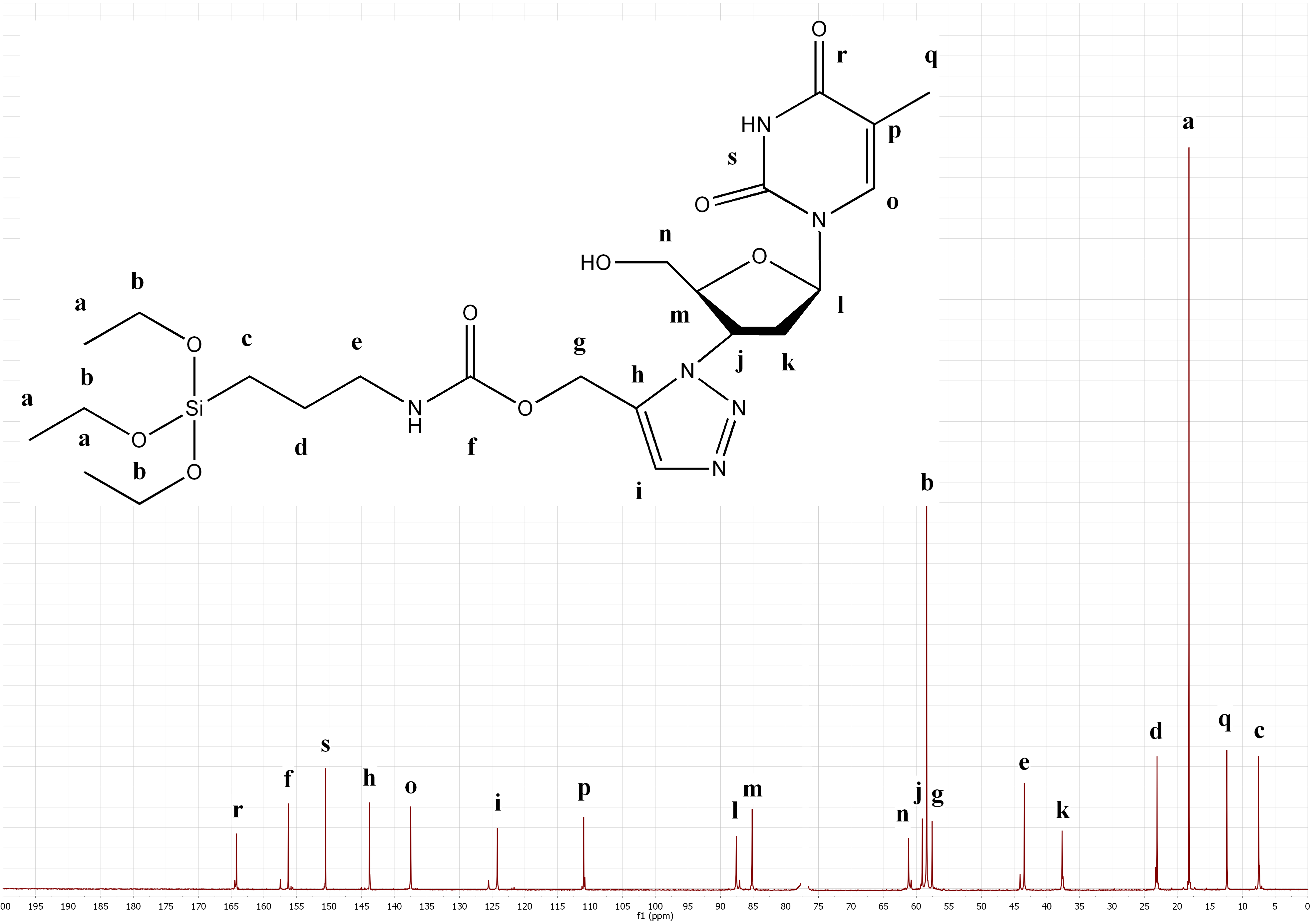

Supplement: S10 Spectrum — (TIF) [file pone.0126251.s010.tif]
